# Maternal Determinants of Birth Weight in Northern Ghana

**DOI:** 10.1371/journal.pone.0135641

**Published:** 2015-08-17

**Authors:** Abdulai Abubakari, Gisela Kynast-Wolf, Albrecht Jahn

**Affiliations:** 1 Community Nutrition Department, School of Allied Health Sciences, University for Development Studies, Tamale, Ghana; 2 Institute of Public Health, Medical Faculty, University of Heidelberg, Germany; McMaster University, CANADA

## Abstract

**Objectives:**

Weight at birth is usually considered as an indicator of the health status of a given society. As a result this study was designed to investigate the association between birth weight and maternal factors such as gestational weight gain, pre—pregnancy BMI and socio—economic status in Northern Ghana.

**Methods:**

The study was a facility-based cross-sectional survey conducted in two districts in the Northern region of Ghana. These districts were purposively sampled to represent a mix of urban, peri—urban and rural population. The current study included 419 mother-infant pairs who delivered at term (37–42 weeks). Mother’s height, pre-pregnancy weight and weight changes were generated from the antenatal records. Questionnaires were administered to establish socio-economic and demographic information of respondents. Maternal factors associated with birth weight were examined using multiple and univariate regressions.

**Results:**

The mothers were generally well nourished before conception (Underweight 3.82%, Normal 57.76%, Overweight 25.06% and Obesity 13.37%) but approximately half of them could not gain adequate weight according to Institute of Medicine recommendations (Low weight gain 49.64%, Adequate weight gain 42.96% and Excessive weight gain 7.40%). Infants whose mothers had excess weight gain were 431g (95% CI 18–444) heavier compared to those whose mothers gained normal weight, while those whose mothers gained less were 479g (95% CI -682– (-276) lighter. Infants of mothers who were overweight and obese before conception were 246g (95% CI 87–405) and 595g (95% CI 375–815) respectively heavier than those of normal mothers, whereas those whose mothers were underweight were 305g (95% CI -565 –(-44) lighter. The mean birth weight observed was 2.98 ± 0.68 kg.

**Conclusion:**

Our findings show that pre-pregnancy body mass index and weight gain during pregnancy influence birth weight. Therefore, emphasis should be placed on counseling and assisting pregnant women to stay within the recommended weight gain ranges.

## Introduction

Birth weight is often considered as an indicator of health status of a given society. Elevated population’s mean birth weight has been linked to good maternity care and healthy living conditions [1]. Birth weight has been shown to be a primary determinant of the chances for survival of a newborn baby [2].

Low birth weight, a proxy measure of intrauterine malnutrition, is a risk factor of fetal and neonatal mortality and morbidity, and chronic diseases which occur later in life such as increased risk of type 2 diabetes, hypertension, and cardiovascular diseases (‘‘the fetal origins hypothesis”) [3–6]. Low birth weight (LBW) has also been associated with deficits in growth and neurocognitive development [7]. In Nigeria, just like in many developing countries, low birth weight is a significant contributor to the overall infant mortality rate and a major factor in the high neonatal mortality rate [8–10].

On the other hand, macrosomia has been implicated in obstetric complications for both mother and baby such as delayed labor, increased need for caesarean delivery, postpartum hemorrhage, birth injuries [5, 11–15] and also cancer development in adulthood [16, 17]. In addition, both LBW and macrosomia have been linked to obesity later in life [18]. However, there seemed to be a distinction between the type of obesity related to LBW and macrosomia respectively; LBW is believed to be concomitant with increased abdominal fat accumulation whereas macrosomia is believed to be concomitant with increased BMI [18, 19].

Both pre-pregnancy body mass index (BMI) and gestational weight gain (GWG) outside of the recommended ranges are associated with low birth weight and macrosomic births. Overweight and obese women are at increased and decreased risks of giving birth to too heavy and too light neonates respectively [20]. Irrespective of pre-pregnancy weight status, women who gain excessive weight during pregnancy are more likely to have macrosomic infants [5, 13, 14, 21]. For normal weight women, weight gain during pregnancy below and above the 1990 and 2009 Institute of Medicine (IOM) recommendations (Table 1) are associated with increased risk of having too light and too heavy neonates respectively [22, 23].

**Table 1:** Institute of Medicine recommendations for total and rate of weight gain during Pregnancy, by pre-pregnancy body mass Index.

Pregnancy BMI (kg/m^2^)	Total weight gain	
	Range (kg)	Mean (range) (kg/wk)
Underweight (<18.5)	12.5–18.0	0.51 (0.44–0.58)
Normal Weight (18.5–24.9)	11.5–16.0	0.42 (0.35–0.50)
Overweight (25.0–29.9)	7.0–11.5	0.28 (0.23–0.33)
Obese (30.0 or higher)	5.0–9.0	0.22 (0.17–0.27)

Source: IOM [23, 52]

Moreover, inadequate prenatal weight gain is shown to be an important risk factor for intra-uterine growth restriction (IUGR), pre-term delivery and low birth weight in infants [24–28]. Obesity and excessive weight gain on the contrary can lead to adverse maternal and fetal outcomes [29, 30]. These have led to suggestions for optimal weight gain to ensure the best outcomes [31].

Other factors that could influence birth weight include pre-pregnancy weight, maternal height, parity, marital status, placental malfunction, smoking, heredity, gender of baby, working hours, and various socio-economic factors [1, 32, 33]. Moreover, a variety of socioeconomic, medical and psychosocial factors are known to be associated with a higher risk of low birth weight [34–37]. In developing countries, the major determinants of LBW babies are genetics, nutrition, low pre-pregnancy weight, short maternal stature, and malaria [38, 39]. A World Health Organization collaborative study of maternal anthropometry and pregnancy outcomes reported that weight gained at 5 or 7 lunar months was the most practical screening for LBW and IUGR [32]. In addition, diseases such as diarrhea, malaria and respiratory infections, which are common in many developing countries, can significantly impair fetal growth when women become infected during pregnancy [34, 35].

Ghana, like many other developing countries, is experiencing a nutrition transition where maternal under nutrition coexists with maternal over nutrition [40, 41]. This is due to changes in lifestyle, diet, urbanization, reduced active commuting to work, use of energy saving devices and increasing sedentary employment that create an ‘obesogenic’ environment [42, 43]. Evidence available shows that in Ghana, the prevalence of overweight and obesity among women of childbearing ages were 26% and 14%in urban women and 14% and 4.2% among rural women respectively [44]. Another study [45] established a prevalence of 31.3% and 37.1% for overweight and obesity respectively among women. However, in the Northern part of Ghana, a lower BMI is always observed [46] compared to the Southern part of Ghana, although the trend in overweight and obesity observed in urban areas in the North is almost comparable to the rest of the country.

Given this paradox with an elevated risk of obesity later in life at both ends of the birth weight spectrum (low and high birth weight), it may be relevant to investigate factors contributing to inadequate or excess birth weight in Northern Ghana where there has been a steady economic growth amidst extreme poverty in the past decade. Besides, this study is the first of its kind to be conducted in Ghana.

The main aim of the study is to assess the association between pre-pregnancy BMI, gestational weight gain (GWG), maternal socio—economic and demographic factors and birth weight.

## Materials and Methods

### Study Site and Design

The study took place in the Northern region, which is one of the ten regions of Ghana. The region is also among the poorest regions in the country. The main occupation of the people in the region is agriculture and related activities. The region has 26 districts, with 24 of them being predominantly rural [47]. This notwithstanding, approximately half of the people live in urban areas with Tamale Metropolis, the regional capital, being the most urbanized city in the region. Illiteracy rate in the region is 62.8% [47].

The study was a facility-based cross-sectional survey. The study took place in two districts in the Northern region of Ghana. The selected districts were Tamale Metropolis and Savelugu—Nanton District. Tamale Metropolis is predominantly urban while Savelugu Nanton District is predominantly rural. These districts were purposively sampled to represent a mix of urban, peri—urban and rural populations, therefore ensuring that the distribution in social groups of the study population can be assumed to be similar to the entire population of the Northern region. A fairly mixed population is also necessary because of the effect of the double burden of malnutrition phenomenon where some populations are over nourished while others are undernourished.

A total of 590 women were recruited in to the study. The study targeted pregnant women and mothers receiving postnatal and child welfare clinic services and have babies who were ≤ 40 days old. Three hundred and sixty (360) of the mothers were approached and those who agreed to participate in the study were asked to sign a consent form. In all 90 women were selected from each of the four hospitals, which included Tamale Teaching Hospital (the largest referral hospital in Northern Ghana), Tamale Central, Tamale West and Savelugu District Hospital using consecutive sampling technique.

**Fig 1:**
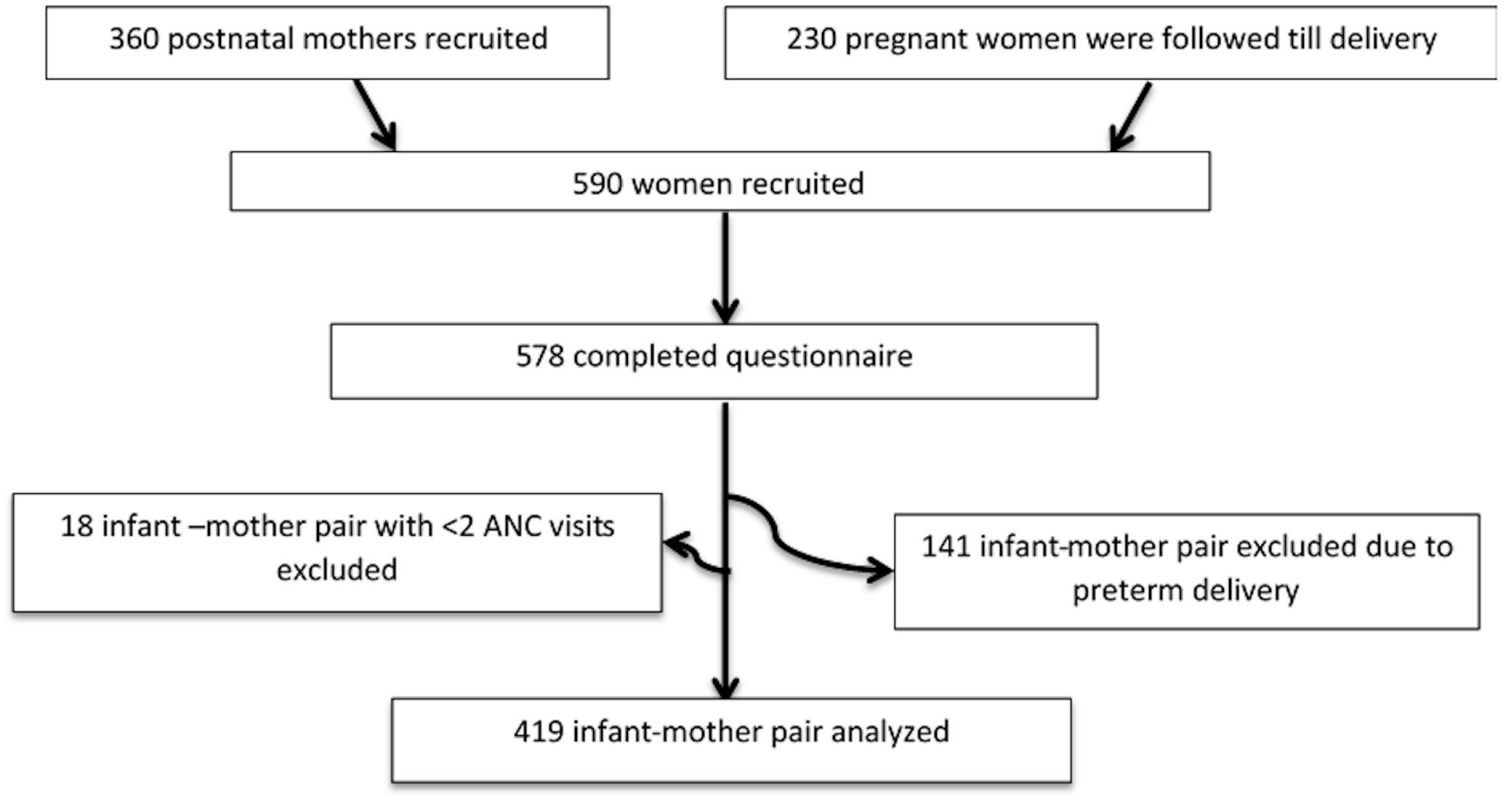
Study profile.

A longitudinal component was conducted at the Tamale Teaching Hospital. The hospital draws its clients across the entire region and beyond. The target group was pregnant women who were in the second trimester of their pregnancy and attending antenatal clinic (ANC) at the Tamale Teaching Hospital. Only singleton pregnancy was considered. Two hundred and thirty (230) pregnant women were selected using consecutive sampling technique and those who agreed to participate in the study were asked to sign a consent form. In all, 578 out of 590 women completed the study questionnaire representing a response rate of 98.0%. For the purpose of this study, 159 mother-infant pairs were excluded due to pre-term delivery (gestational age <37 weeks = 141). Eighteen mother-infant pairs who had less than three ANC visits before delivery were also excluded because of late initiation of first booking for ANC (Fig 1). Therefore, analysis was performed on 419 respondents. The weight of the mother at first booking for ANC during first trimester is considered as an appropriate proxy for pre-pregnancy weight [48, 49] since weight gain in the first trimester is low (approximately 1kg) [23].

Structured questionnaires were administered to the mothers by a team of trained interviewers. The questionnaires collected information on demographic and socio-economic characteristics of mothers. Information on health status, ANC attendance, pre-pregnancy weight and weight per ANC visit, height of mother, birth weight and gestational age was retrospectively recorded from the ANC booklet for the nursing mothers whereas the remaining information of the pregnant women was recorded as and when they came for ANC visit until delivery.

Birth weight was analyzed as a continuous and categorical variable. When describing the prevalence of low birth weight and macrosomic births, low birth weight was defined according to WHO criteria as <2.5 kg [50] and macrosomic births as ≥90^th^ percentile. The determinants studied were GWG, pre-pregnancy BMI, pre-existing disease (such as hypertension = 8, sickle cell = 4, heart diseases = 3, TB = 2 and diabetes = 1 were collapsed into one variable called pre—existing disease due to the small prevalence), marital status, maternal age, and sex of the infant.

The socio-economic status of the household was determined using the household wealth index as a proxy, which was derived from household assets such as availability of potable water, electricity, television, refrigerator, motorcycle, car/tractor/truck, washing machine, gas cooker and livestock. These facilities/durable goods are often regarded as modern goods that have been shown to reflect household socio-economic status [51]. Principal component Analysis (PCA) was used to compute the household wealth index in STATA. This was further ordered/categorized in to three using tertiles. The first tertile represented the lower socio-economic group; the middle tertile represented the middle class and the third tertile represented higher socio economic class.

Gestational weight gain (GWG) was calculated by subtracting maternal weight measured at the end of gestation from the weight measured at first booking for antenatal care during first trimester (a proxy of pre-pregnancy weight). GWG was categorized as inadequate, normal or excessive according to the recommendations from IOM, which were developed to optimize the growth of the fetus and also to prevent mothers from gaining too much weight during pregnancy. The recommendations state that underweight women (BMI < 18.5) should gain 12.5–18.0 kg, normal weight women (BMI 18.5–24.9) should gain 11.5–16.0 kg, overweight women (BMI 25–29.9)should gain 7.0–11.5kg, and obese women (BMI > 29.9) should gain at least 6.8kg [23, 52] (Table 1).

### Data Analysis

The data was entered using Epi Info version 4.1 and later imported to STATA 12.1 for analysis. Descriptive statistics include means and standard deviations (S.D.) for continuous variables and frequency distributions for categorical variables. Relationships between categorical variables and birth weight means were determined by independent *t*-test. One-way ANOVA was used to compare means where more than two categories were formed. Multiple testing was controlled for by the use of Bonferroni corrections.

The influence of the different determinants on birth weight was assessed by multiple regression analysis. The first procedure in the analysis was to screen all potential determinants individually for relationship with the dependent variable. Variables, which showed a p-value ≤0.3 in the univariate models, were selected. Weight gain and BMI as continuous traits were also included in the model to test the power of the model but due to multicolinearity BMI as a continuous trait was excluded in the final model (Tables A and B in S1 File).

It is important to note that comparing continuous and categorical variables is not straightforward since the value for continuous variable denotes the amount of S.D. change in *y* for a S.D. change in *x*, while the value for the categorical variable *x* denotes the amount of S.D. change in *y* for a change between categories (e.g. rural to urban). This notwithstanding, it is possible to compare categorical and continuous variables so far as these problems are kept in mind when interpreting the results.

Consistency and plausibility checks were done after the data entry to ensure that errors were reduced. We checked multicollinearity and singularity among independent variables. We also checked normality, linearity, and homoscedasticity by using residual scatter plots. P-value<0.05 at 95% confident level was considered statistically significant.

The mothers were asked to sign a written consent form. For those who could not read and write, this was done through an interpreter. The guardians or parents were asked to sign a written parental or guardian form on behalf of the minors while the minors were asked to sign an assent form. The Ethics committees of Navarongo Health Research Center (Ref. No: App/MatNut/01/2014) (S1 Text) and the University of Heidelberg institutional review board approved this study. The study took place between February and August 2014.

## Results

Majority of the women were Dagombas (78.28%), 4.30% were Gonjas, 3.5% were Mampurisi and 13.92% were other ethnic groups (For example Farafaras, Mooses, Hausas, Bimobas, Konkombas, Asantis, Ewes, Fulanis etc). Almost all the respondents (99.76%) were married. Majority were also Muslims (94.03%) while the remaining 5.97% were Christians. Also 57.76% had normal pre-pregnancy BMI whereas 3.82% were underweight, 25.06% were overweight and 13.37% were obese. Most of the women had some formal education (63.25) while 36.75% had no formal education (Table 2).

**Table 2:** Maternal and infant characteristics and birth weight.

Variables	Categories	N/%	Mean ± SD (kg)	P—value
**Weight gain**	**Adequate**	**180(42.96%)**	**3.17±0.69**	**< .0001** ^**a**^ ^,^ ^**b**^
	**Low weight gain**	**208(49.64%)**	**2.95±0.68**	
	**Excess weight gain**	**31(7.40%)**	**3.64±0.77**	
**Pre—pregnancy BMI**	**Normal**	**242(57.76%)**	**2.69±0.43**	**< .0001** ^**a**^ ^,^ ^**c**^
	**Underweight**	**16(3.82%)**	**2.28±0.42**	
	**Over weight**	**105(24.58%)**	**3.26±0.64**	
	**Obese**	**56(13.37%)**	**3.76±0.81**	
**Sex**	**Male**	**218(52.03%)**	**3.03±0.68**	**0.0106** ^**t**^
	**Female**	**201(47.97%)**	**2.87±0.68**	
**Hemoglobin level (g/d)**	**Below normal**	**195(46.50%)**	**2.95±0.63**	**0.0137** ^**t**^
	**Normal**	**224(53.50%)**	**3.03±0.72**	
**Age of mother (years)**	**16–20**	**35(8.35%)**	**2.78±0.39**	**0.0425** ^**a**^ ^,^ ^**d**^
	**21–30**	**224(53.46%)**	**2.93±0.70**	
	**>30**	**117(27.92%)**	**3.01±0.79**	
**Diarrhea episode**	**Had no diarrhea**	**361(86.16%)**	**2.98±0.69**	**0.0316** ^**t**^
	**Had diarrhea**	**58(13.84%)**	**2.78±0.70**	
**Pre—existing disease**	**No pre—existing disease**	**401(95.70%)**	**2.94±0.68**	**0.0191** ^**t**^
	**Had pre—existing disease**	**18(4.30%)**	**3.32±0.72**	
**Mother’s location**	**Urban**	**349(83.29%)**	**3.03±0.68**	**< .0001** ^**t**^
	**Rural**	**70(16.71%)**	**2.60±0.58**	
**Educational status**	**Had some education**	**265(63.25%)**	**3.03±0.68**	**0.0057** ^**t**^
	**Had no education**	**154(36.75%)**	**2.83±0.51**	
**Mother’s occupation**	**Informal**	**265(63.25%)**	**2.94±0.71**	**0.2105** ^**a**^ ^,^ ^**e**^
	**House wife**	**90(21.48%)**	**2.90±0.64**	
	**Formal sector**	**64(15.06%)**	**3.10±0.63**	
**Socio—economic status**	**Middle class**	**149(35.56%)**	**2.99±0.68**	**0.0044** ^**a**^ ^,^ ^**f**^
	**Low class**	**126(30.07%)**	**2.80±0.60**	
	**Upper class**	**144(34.37%)**	**3.06±0.74**	
**Usage of intermittent preventive treatment**	**Did not take IPT**	**156(37.23%)**	**2.92±0.64**	**0.5650** ^**a**^ ^,^ ^**g**^
	**One dose**	**112(26.73%)**	**3.02±0.78**	
	**Two doses**	**108(25.78%)**	**2.97±0.66**	
	**Three doses**	**43(10.26%)**	**2.90±0.64**	
**Religion**	**Muslim**	**394(94.03%)**	**2.94±0.68**	**0.1286** ^**t**^
	**Christian**	**25(5.97%)**	**3.16±0.66**	
**Ethnicity**	**Dagombas**	**328(78.28%)**	**2.94±0.68**	**0.3172** ^**a**^ ^,^ ^**h**^
	**Gonja**	**18(4.30%)**	**3.01±0.71**	
	**Mampurisi**	**15(3.5%)**	**2.74±0.82**	
	**Other ethnic groups**	**58(13.92%)**	**3.08±0.69**	

^a^ one-way anova

^t^ independent t-test

^b^ Inadequate, Normal excessive weight gain

^c^ <18.5, 18.5–24.99, >25<30, >30

^d^ 16–20, 21–30, >30

^e^ Informal sector, Housewife, Formal sector

^f^ Low income Household, Middle income household, Upper income household

^g^ Did not take IPT, one dose, two doses, Three doses

^h^ Dagombas, Gonjas, Mampurisi, other ethnic groups (Farafaras, Fulanis, Ashantis, Mooses etc)

Moreover, the mean weight gained during the entire period of the pregnancy was 7.35 ± 4.28kg. Approximately half of the women could not gain within the recommended weight (49.64%) while 42.96% gained within the recommended weight and 7.40% gained more than the recommended weight (Table 2).

### Differences in Birth Weight

Significantly higher birth weights were observed in infants whose mothers gained excessive weight (0.47±0.08 kg), were overweight (0.57±0.21 kg), were obese (1.12±0.17 kg) and were urban dwellers (0.43±0.10 kg), among male infants (0.16±0.00 kg), those who had pre-existing disease (0.38±0.03 kg) and those whose mothers had some formal education (0.2±0.19 kg) (Table 2). On the other hand, significantly lower birth weights were observed in infants with mothers who gained inadequate weight (-0.51±0.18 kg), were underweight (-0.40±0.01 kg), those whose mothers had lower hemoglobin level (-0.16±0.09 kg) and those whose mothers had episode of diarrhea during pregnancy (-0.20±0.01 kg) (Table 2).

The mean birth weight was 2.98 ± 0.68 kg (Table 3). The majority of the infants had normal birth weight (62.69%). Out of the remaining, 26.01% had low birth weight while 11.69% were macrosomic.

**Table 3:** Maternal and infant characteristics.

Variables (continuous)	Mean±S.D	Median	Ranges
Birth weight (kg)	2.98 ± 0.68	2.9	1.6–5.2
Gestational age (weeks)	38.7 ± 1	39	37–42
Maternal age (years)	28.0 ± 5	27	16–41
Maternal pre-pregnancy weight (kg)	63.1 ± 12.70	60.0	42–119
Maternal height (cm)	160.0 ± 6.0	159.0	144–190
Pre-pregnancy BMI (Kg/m^2^)	24.64 ± 4.75	23.71	15.9–47.0
Weight gain (kg)	7.35 ± 4.28	7	0–22
Early pregnancy Haemoglobin level (g/dl)	11.0 ± 1.43	11.1	5.2–15.6
ANC utilisation	4.6 ± 1.0	4.0	3–6

### Univariate Regression Analysis

In the univariate regression model, pre-pregnancy BMI classified as underweight, normal, overweight and obese, gestational weight gain according IOM classification (low weight gain, adequate and excessive weight gain), location of the mother (rural/urban), sex of infant, hemoglobin level during early pregnancy, pre-existing disease (hypertension, sickle cell, TB, heart disease and diabetes), ANC utilization and episode of diarrhea during pregnancy were significantly associated with birth weight (Table 4).

**Table 4:** Determinants of birth weight (Univariate regression).

Variables	Categories	ß	P-value	95%CI
Weight gain according to IOM	Adequate weight gain	Ref.		
	Low weight gain	-507	< .0001	-630 –(-385)
	Excess weight gain	467	< .0001	233–700
Total weight gain		18	< .0001	2–33
Pre-pregnancy BMI	Normal	Ref.		
	Under weight	-404	0.005	-682 –(-126)
	Over weight	574	< .0001	448–800
	Obese	1,079	< .0001	919–1,239
Gestational age (weeks)		179	< .0001	129–230
Early pregnancy haemoglobin		84	< .0001	38–130
Age of mother		15	0.02	2–27
Total number of ANC visit		85	0.016	15–154
Sex	Male	Ref.		
	Female	-170	0.011	-301 –(-39)
Mother’s location	Urban	Ref		
	Rural	-424	< .0001	-596 –(-253)
Pre-existing disease	No pre-existing disease	Ref.		
	Had pre-existing disease	386	0.019	63–709
Diarrhoea episode	Had no diarrhoea	Ref		
	Had diarrhoea	-209	0.034	-401 –(-16)
Mother’s occupation	Informal sector	Ref.		
	House wife	-39	0.638	-203–125
	Formal sector	147	0.124	-40–334
Socio-economic status	Middle class	Ref.		
	Low class	-192	0.02	-353 –(-31)
	Upper class	74	0.347	-81–230
Educational status	Had secondary education	Ref.		
	Had no formal education	-189	0.017	-343 –(-34)
	Had basic education	-15	0.887	-235–203
	Had tertiary education	19	0.843	-177–216
Religion	Muslims	Ref		
	Christians	-215	0.128	-492–62
Ethnicity	Dagombas	Ref.		
	Gonja	67	0.685	-260–393
	Mampurisi	-204	0.260	-559–151
	Other ethnic groups	132	0.177	-60–324

Ref. = Reference

### Multiple Regression Analysis

More covariates were included in the multiple regression model. The final model explained 49.0% of the variance in birth weight. Excessive weight gained during pregnancy, overweight, obesity and gestational age were positively associated with birth weight while the location of mother (rural), sex of the baby (female), inadequate weight gain, weight gain as a continuous trait and underweight were negatively associated with birth weight (Table 5).

**Table 5:** Determinants of birth weight (Multiple regression).

Variables	Categories	ß	P-value	95% CI
Weight gain according to IOM	Adequate weight gain	Ref.		
	Low weight gain	-479	< .0001	-682 –(-276)
	Excess weight gain	431	0.001	18–682
Total weight gain		-36	0.004	-61 –(-11)
Pre-pregnancy BMI	Normal	Ref.		
	Under weight	-305	0.022	-565 –(-44)
	Over weight	246	0.003	87–405
	Obese	595	< .0001	375–815
Early pregnancy haemoglobin		25	0.162	-10–60
Age of mother		-2	0.643	-12–7
Total number of ANC visit		-25	0.411	-87–36
Gestational age		103	< .0001	61–145
Sex	Male	Ref.		
	Female	-176	0.001	-275 –(-77)
Diarrhoea episode	Had no diarrhoea	Ref		
	Had diarrhoea	-193	0.009	-337 –(-49)
Mother’s location	Urban	Ref		
	Rural	-206	0.004	-348 –(-65)
Pre-existing disease	No pre-existing disease	Ref.		
	Had pre-existing disease	212	0.090	-33–457
Socio-economic status	Middle class	Ref.		
	Low class	-49	0.450	-78–176
	Upper class	-11	0.875	-155–132
Educational status	Had secondary education	Ref.		
	Had no formal education	-17	0.788	-145–110
	Had basic education	49	0.560	-117–216
	Had tertiary education	-26	0.742	-183–130

Ref. = Reference

Model Power (R^2^) = 0.49

In addition to this, the ß–coefficient shows that infants whose mothers had excessive weight gain were 431g heavier compared to those who gained adequate weight. Similarly, infants whose mothers were overweight and obese before pregnancy or during the early stages of pregnancy were 246g and 595g respectively heavier compared to those whose mothers had normal weight. In the same vein, for every one-week increase in gestational age the infant was 103g heavier (Table 5). On the other hand, infants whose mothers gained less weight and were underweight were 479g and 304g respectively lighter compared to those who were born by mothers who gained adequate weight or had normal weight. Likewise, infants who were born by rural dwellers were 206g lighter as compared to those born by urban dwellers, and those whose mothers had episode of diarrhea during pregnancy were also 193g lighter as compared to those who did not have any episode of diarrhea. Female infants were also 176g lighter as compared to their male counterparts (Table 5).

## Discussion

The mothers in this study were generally well nourished before conception or during the early stages of the pregnancy (underweight 3.82%, normal 57.76%, overweight 25.06%and obesity 13.37%), but approximately half of them could not gain adequate weight according to IOM recommendations (Low weight gain 49.64%, Adequate weight gain 42.96% and Excessive weight gain 7.40%). They also had infants who had IUGR, as the proportion of low birth weight was 26.01%.

The most important determinants identified in this study were pre-pregnancy BMI, GWG, gestational age, sex of infant, mother’s location (rural/urban) and episode of diarrhea during pregnancy. The classifications of GWG according to the IOM recommendations were shown to be strongly associated with birth weight, with infants of mothers gaining excessive weight being 431g heavier than those with mothers who gained adequate weight while those of mothers who gained less were 479g lighter. Similarly infants of mothers who were overweight and obese were 246g and 595g heavier than those whose mothers had normal BMI while those whose mothers were underweight were 304g lighter.

Most of the studies on birth weight have investigated risk factors of either low or high birth weight [1, 13, 53–56] rather than analyzing birth weight as a continuous variable. In Africa and Ghana in particular, there appeared to be no study to the best of the authors’ knowledge analyzing birth weight as a continuous variable. Therefore, it is important to bear in mind that comparison of findings of this study to others may not be straightforward.

Nevertheless, the findings of one study that analyzed birth weight as a continuous variable and also investigated a large number of determinants were largely consistent with the findings of this study. They found that infants with mothers who gained excessive weight were heavier than those with mothers who did not gain adequate weight [57]. This finding buttresses the importance of optimal nutrition during preconception and pregnancy [58]. They also observed that 25% of the variance in birth weight was explained by the predictors studied whereas in this study, 49% of the variance in birth weight was explained by the determinants studied. This could be due to the fact that other variables such as early pregnancy hemoglobin, ANC utilization, socio-economic status of respondents and diarrhea episodes during pregnancy, which are considered to be important predictor of birth weight especially in developing countries [36, 58, 59] were included in the present study.

Beside this, the findings of one other study [37] in low—income American natives were in some aspect consistent with this study but not consistent in others. For instance, it shows that inadequate weight gain was associated with low birth weight and the same association was amazingly found for excessive weight gain. Perhaps risky behaviors such as smoking and alcohol consumption are more prevalent in low-income American natives than mothers in the present study. These risky behaviors could be important determinants of infant weight at birth in low-income American natives than weight gain and therefore, could be responsible for May’s [37] observation. The study subjects of the present study neither smoked nor consumed alcohol.

However, the impact of excessive weight gain and inadequate weight gain conform to other studies [5, 13, 60, 61] analyzing low and high birth weight separately. For example, a review conducted in 2000 on fetal growth concluded that there was a high risk of low birth weight in infants with mothers whose GWG was below the IOM recommendation and a greater risk of high birth weight with GWG higher than IOM ranges [61]. Stottland et al [5] also show in a retrospective cohort study that weight gain below IOM recommendation was associated with increased odds for small for gestational age, while weight gain above the IOM recommendation was associated with increased odds for infant born large for gestational age.

In another study [13], investigators concluded that women who gained more than IOM recommendation were three times more likely to have infants with high birth weight after adjusting for other factors. Another study conducted in Pakistan concluded that inadequate weight gain had higher odds for low birth weight, while those who gained excessive weight had high odds for large for gestational age [62].

Many of the determinants identified in this study (e.g. pre-pregnancy BMI, GWG, infant sex and location of the mother) have consistently been found to be significant predictors in studies of either low or high birth weight [53–55, 63, 64]. However, the effect of maternal age, marital status, and chronic diseases such as diabetes and hypertension on birth weight is less consistent in literature [55, 63, 65].

Also the mean birth weight found in this study is not very different from other studies conducted in the Northern region of Ghana especially Tamale. For example, one study conducted in 2012 at the Tamale Teaching Hospital found a mean birth weight of 2.85±0.5 kg [66], which is only slightly lower than what we observed in this study. In a well-fed population such as women delivering in private hospitals in the Northern region of Ghana, the mean birth weight was found to be 3.2kg, which is higher than the one observed in this study.

In the final regression model, 49% of the variance in birth weight was explained by the determinants investigated. This fits well with studies that have shown that 40% of birth weight is explained by genetic and 60% by environmental factors [67]. In addition to this, Kramer [63] concluded that a large number of factors theoretically could influence birth weight while Ohlin & Rossner [68] indicated that each of the factors has a rather small individual impact. This could therefore explain the findings of fairly modest impact of the determinants in this study and probably others.

With regards to the nutritional status of the mothers, it was generally observed that the women were well nourished before conception or during the early stages of pregnancy, but approximately half of them could not gain weight according to the recommended. This finding appeared to corroborate other studies conducted in western countries. For example, one study conducted in Germany between 1995 and 2000 reported that short and heavy women had lower weight gain than tall and thin women during pregnancy [69]. Another study conducted in the United States between 2004 and 2005 showed that weight gain during pregnancy decreases with increasing BMI [70]. Could this be the same reasons for low weight gain among pregnant women in Northern Ghana or other factors such as inadequate food intake due to pregnancy related behaviors such as ‘pica’ practice (perverted appetite for substances not fit as food or of no nutritional value) which is common in Ghana [71], or cultural factors such as the belief that if you eat well and gain weight during pregnancy your baby becomes big and you may have difficult labor or cesarean section? Further studies are needed in order to explain why approximately half of the mothers could not gain adequate weight and also the high proportion of low birth weight recorded although the mothers were generally observed to be well nourished before conception.

The strength of this study was that GWG was calculated using the measured weight at the end of gestation at maternity ward admission. This procedure ensures that the women did not gain additional weight that was not accounted for. However, an important limitation was that the study used routine data recorded by health professionals during pregnancy. Therefore, measurements errors concerning readings or recordings of parameters such as height, weight and hemoglobin level and other indices were likely to occur. However, the effect of these errors was random and unlikely to affect our findings. Using weight at first booking for ANC in the first trimester as a proxy for pre-pregnancy weight was another limitation as it could lead to classification bias.

## Conclusion

On the whole, pre-pregnancy BMI and GWG were found to be the most important determinants of birth weight after controlling for gestational age. In most developing countries especially in Sub-Saharan African countries, counseling on appropriate weight gain in pregnancy during antenatal clinics and optimal nutrition before pregnancy for prospective mothers are mostly ignored. Since GWG is a modifiable risk factor, and recommendations exist for different BMI groups, it is important that emphasis should be placed on counseling and assisting pregnant women to stay within the recommended GWG range.

Discussions about weight gain are especially important since approximately half of the mothers in this study had low GWG, which is associated with high risk of low birth weight. Beside this, a significant number of the women also had GWG above the recommended ranges and this is also considered to be a risk factor for macrosomic birth. A significant proportion of the pregnant women were over-nourished, which is a clear sign of the double burden of malnutrition currently experienced by developing and transition countries. Our findings especially on weight gain during pregnancy call for more research on factors that influence weight gain during pregnancy in Ghana.

## Supporting Information

S1 File Tables A and B Test for Multicolinearity (VIF values). (DOCX)

S1 Text Ethical Clearance Certificate. (ZIP)
