# Supplementary material for: Maternal Determinants of Birth Weight in Northern Ghana
Source: PLoS One. 2015 Aug 17;10(8):e0135641. doi: 10.1371/journal.pone.0135641 (PMC4539219; doi:10.1371/journal.pone.0135641)
Supplement: S1 File — (DOCX) [file pone.0135641.s001.docx]

**Table A. Test for Multicolinearity (VIF values)**

estat vif

Variable VIF 1/VIF

wightgain_1

Low weight gain 4.39 0.227902

Excess weight gain 1.87 0.535692

bmi2

Underweight 1.23 0.811881

Overweight 3.15 0.317681

Obese 6.16 0.162344

anc_visits 1.43 0.698675

Ageofmother 1.16 0.864328

Hb 1.08 0.927319

Gestation 1.13 0.885835

0.Sex 1.03 0.972646

0.Location_1 1.19 0.841049

1.Havdaria 1.04 0.959691

1.chronicd~s 1.06 0.946579

Education_1

3 1.60 0.623101

4 1.25 0.798307

5 1.40 0.714836

pcathre

Low 1.44 0.693127

High 1.52 0.656523

bmi continouos trait 6.28 0.159133

weightgain 4.63 0.215996

Mean VIF 2.20

**Table B. Test for Multicolinearity (VIF values)**

estat vif

Variable VIF 1/VIF

wightgain_1

Low weight gain 4.34 0.230428

Excess weight gain 1.85 0.541044

bmi2

Underweight 1.07 0.935720

Overweight 2.00 0.499866

Obese 2.36 0.423695

anc_visits 1.43 0.699156

Ageofmother 1.15 0.867899

Hb 1.07 0.932983

Gestation 1.13 0.886516

0.Sex 1.03 0.973461

0.Location_1 1.19 0.841455

1.Havdaria 1.03 0.969507

1.chronicd~s 1.06 0.947245

Education_1

3 1.59 0.627852

4 1.25 0.798332

5 1.39 0.720478

pcathre

Low 1.44 0.693239

High 1.52 0.657142

Weight gain continuous trait 4.53 0.220793

Mean VIF 1.71
