# Supplementary material for: Maternal Determinants of Birth Weight in Northern Ghana
Source: PLoS One. 2015 Aug 17;10(8):e0135641. doi: 10.1371/journal.pone.0135641 (PMC4539219; doi:10.1371/journal.pone.0135641)
Supplement: S1 Text — (ZIP) [file pone.0135641.s002.zip › Ethical clearance certificate/Maternal Nut - Abdulai.pg1-1.pdf]

In case of reply the
number and date of this
letter should be quoted.

My Ref. App/MatNut/01/2014
Your Ref:

Navrongo Health Research Centre
Institutional Review Board
Ghana Health Service
P. O. Box 114
Navrongo, Ghana

Tel/Fax: +233-3821-22348
Email: irb@navrongo-hrc.org

31st January, 2014

Mr. Abdulai Abubakari
University of Heidelberg
Institute of Public Health (PhD Student)
Bergheimer Str. 20, 69115 Or
Im Neuenheimer Feld 674/04-14-0
69120
Heidelberg, Germany

ETHICS APPROVAL ID: NHRCIRB174

Dear Mr. Abubakari

Approval of protocol titled: ***"The role of maternal nutrition and other factors in foetal growth"***

Following your satisfactory address of the concerns raised by the NHRCIRB expedited review of the above-mentioned protocol, the Board is pleased to grant you approval.

The documents that were reviewed and approved include the following:

- Protocol submission form
- Study protocol version 2.0 dated 23/12/2013
- Consent form – English Version 2.0 dated 23/12/2013 for Adults
- Assent form – English Version 1.0 dated 21/01/2014 for 14 – 17 years of age
- Parental consent for adolescents - English Version 1.0 dated 21/01/2014 for 14 – 17 years of age

Please note that any amendment to this approved protocol must receive ethical clearance from the NHRCIRB before its implementation.

The Board should be notified about the actual start date of the project and would expect a report on your study, annually or at the close of the project, which ever comes first. Should you require
