# Supplementary material for: Maternal Determinants of Birth Weight in Northern Ghana
Source: PLoS One. 2015 Aug 17;10(8):e0135641. doi: 10.1371/journal.pone.0135641 (PMC4539219; doi:10.1371/journal.pone.0135641)
Supplement: S1 Text — (ZIP) [file pone.0135641.s002.zip › Ethical clearance certificate/Maternal Nut - Abdulai.pg2-1.pdf]

a renewal of your approval, the report should be submitted two (2) months before the expiration date.

You are also to note that this approval expires on 30th January, 2015.

The Board wishes you the best in this study.

Sincerely,

Dr. (Mrs.) Nana Akosua Ansah
(Vice Chair, NHRCIRB)

Cc: The Director
NHRC, Navrongo
